# Morphometrics of Facies Patellaris Femoris in Dry Bones

**DOI:** 10.7759/cureus.38839

**Published:** 2023-05-10

**Authors:** Feyza Aksu, Ramazan Fazil Akkoc

**Affiliations:** 1 Department of Anatomy, Faculty of Medicine, Firat University, Elazig, TUR

**Keywords:** morphometry, distal femur anatomy, trochlear index, trochlea, facies patellaris

## Abstract

Introduction: The aim of this study was to investigate the morphological features of the distal femur, with a specific focus on the facies patellaris femoris.

Methods: A total of 45 dry femurs from adult individuals (24 right, 21 left) were used for the study. Measurements were taken using a calibrated digital vernier caliper and a contour gauge.

Results: Anteroposterior (AP) measurements were taken for the medial and lateral condyles of the femur, as well as the articular surfaces of the facies patellaris, sulcus height (51.186±3.81mm), trochlear depth (7.436±1.19mm), and trochlear index (2.295±0.06mm). The results showed that the width of the facies patellaris had a significant positive correlation with the trochlear depth and trochlear index. The length of the facies patellaris was positively correlated with the AP length of the medial condyle and sulcus height, although it was not statistically significant. Additionally, there was a statistically significant positive correlation between the length, width, and medial and lateral articular surfaces of the facies patellaris (p<0.005).

Conclusion: Understanding the relationship between the morphometry of the medial and lateral condyles of the distal femur and the morphometry of the facies patellaris, sulcus height, trochlear depth, and trochlear index and examining the anatomy of the distal femur and patella in individuals are crucial factors for determining appropriate medical treatment and implant selection and compatibility. The findings of this study are expected to contribute to clinicians' interventions in this region (total knee arthroplasty/replacement operation etc.). These data can also be used by implant designers and forensic experts during investigations.

## Introduction

Bones exhibit unique external appearances, with distinct anatomical formations and surface features. There are variations in the size and shape of pits, elevations, and articular surfaces of different individuals' bones [1,2].

The femur, the longest and strongest bone in the body, is composed of three segments: the upper end (proximal), shaft (corpus), and lower end (distal). The lower end is wider in all dimensions compared to the upper end. On both sides of the broader lower end, there are prominent formations known as lateral condyle and medial condyle. The articular surfaces of these condyles converge anteriorly to form the facies patellaris, which is divided into outer (lateral) and inner (medial) facets by a groove called "trochlea" [3]. The posterior surface of the patella rests on this groove, which has a vertical edge. During flexion and extension of the knee joint, the patella glides over the facies patellaris [4].

The flexion and extension movements of the patellofemoral joint are complex and dynamic processes that involve both osseous structures and soft tissue balance. The lower end of the femur, facies patellaris, and patella are static forces that play a crucial role in the management of articular pathology, and their harmonious interaction is of utmost importance [5].

In regions where diverse populations reside, the morphometry of this region should be prioritized in total knee arthroplasty/replacement operation that affects the condyles and facies patellaris at the lower end of the femur. Ensuring harmony between the facies patellaris and patella is essential to prevent an increase or decrease in pressure in the retropatellar area, which may occur after prosthetic operations [6].

Therefore, having knowledge of the normal morphometry of the lower end of the femur, specifically the facies patellaris and condyles, is crucial for designing prostheses that are most appropriate and effective for the regional population.

The aim of this study was to conduct morphometric measurements of the facies patellaris and condyles of femurs in the anatomy laboratory of Fırat University Faculty of Medicine, with the goal of contributing to both the literature and clinical practices.

## Materials and methods

**Figure 1:**
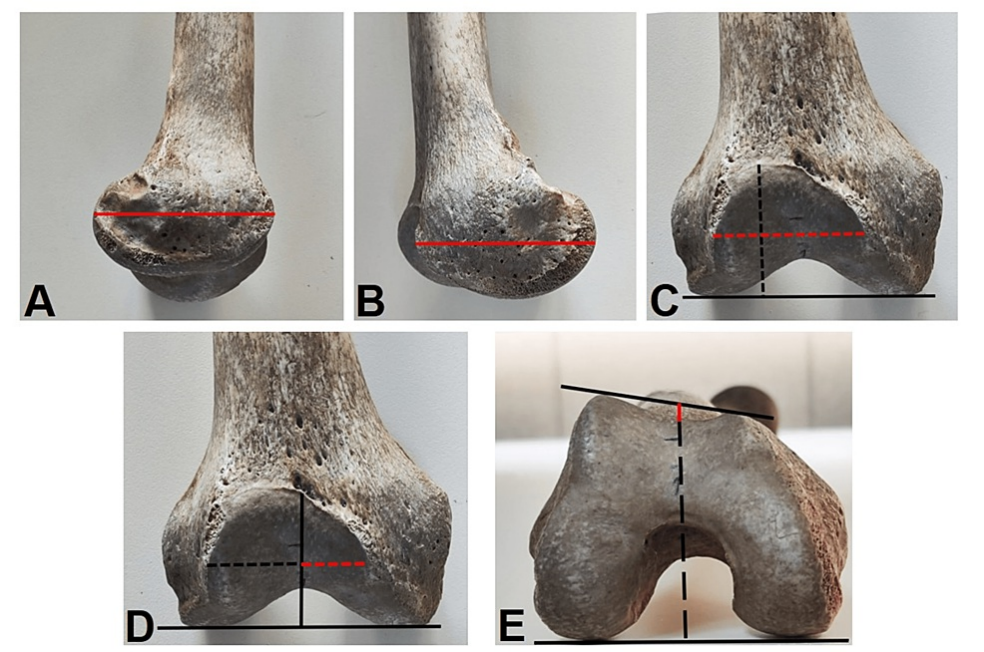
Parameters of measurements A: Lateral condyle AP length, B: Medial condyle AP length, C: Dashed black line: Facies patellaris length and dashed red line: Facies patellaris width, D: Dashed red line: Facies patellaris medial articular surface and dashed black line: Facies patellaris lateral articular surface E: Dashed black line: Sulcus height and red line: Trochlear depth AP: Anteroposterior

This study was initiated based on the decision of the Fırat University Non-Interventional Research Ethics Committee, dated November 3, 2022, with session number 2022/13-17. Forty-five dry bones (femurs) obtained from adult human specimens in the Department of Anatomy at Fırat University Faculty of Medicine were included in the study. The femurs used in the study were anatomically intact and complete. Morphometric measurements for the distal femur and trochlea were conducted following the previously described methods [7-9]. The definitions of the measurements are provided in Table 1 (Figure 1).

**Table 1:** Measurement definitions for the distal femur AP: Anteroposterior

Measurement	Description
Medial condyle AP length	Distance between the most anterior and posterior aspects of the medial condyle
Lateral condyle AP length	Distance between the most anterior and posterior aspects of the lateral condyle
Sulcus height	Distance between the ground and the femoral sulcus in the ground contact position of the posterior aspects of the medial and lateral condyles
Trochlear depth	Distance between the ventral border of the medial and lateral condyles and the deepest point of the femoral sulcus
Trochlear index	The result obtained by dividing the sum of the medial condyle AP length and the lateral condyle AP length by the sulcus height
Facies patellaris length	Distance between the upper border of the facies patellaris and femoral sulcus
Facies patellaris width	Maximum distance between the medial and lateral aspects of the facies patellaris
Facies patellaris medial articular surface	Distance between femoral sulcus and the medial end of the facies patellaris
Facies patellaris lateral articular surface	Distance between femoral sulcus and the lateral end of the facies patellaris

**Figure 2:**
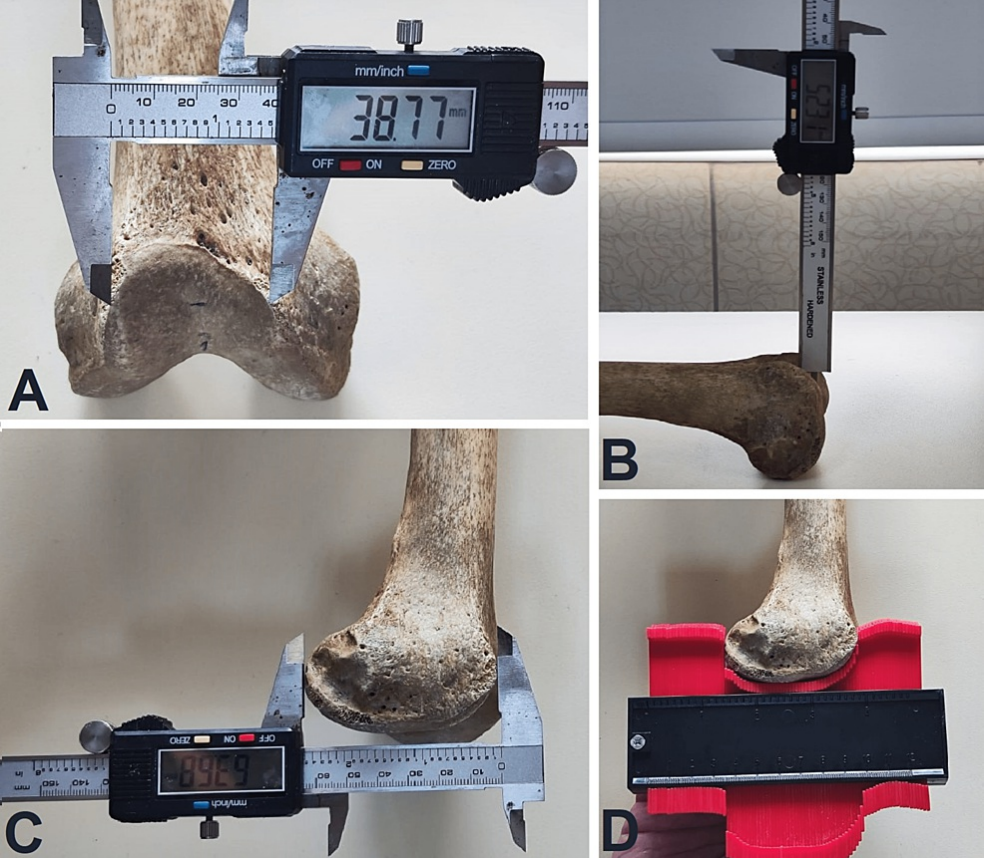
Morphometric measurements A: Maximum distance between the medial and lateral aspects of the facies patellaris, B: Measurement of the sulcus height with a digital vernier caliper, C: Measurement of the lateral condyle AP diameter with a digital vernier caliper, D: Determination of the lateral condyle AP diameter with a contour gauge AP: Anteroposterior

All measurements were conducted using a calibrated digital vernier caliper with a measurement range of 0-150 mm, a resolution of 0.01 mm, and an accuracy of ± 0.02 mm, along with a contour gauge (Figure 2). There was no statistically significant difference between the two researchers' measurements. All measurements were taken on a flat surface.

Statistical analysis

Statistical analysis of the data was performed using IBM SPSS Statistics for Windows, Version 22 (Released 2013; IBM Corp., Armonk, New York, United States). Categorical measurements were presented as numbers and percentages, while measurements were reported as mean and standard deviation. Normal distribution assumption was tested using the Kolmogorov-Smirnov test. The independent sample t-test was employed for intergroup comparison of measurements. Pearson's correlation coefficient and the corresponding p-value were calculated to examine the interaction between measurements. The level of statistical significance was set at 0.05 for all tests.

## Results

Out of the 45 dry bones examined, 24 (53.3%) were right femurs and 21 (46.7%) were left femurs. Table 2 presents the mean, minimum, maximum, and standard deviation values for all measurements.

**Table 2:** Morphometric measurement values of the distal femur and facies patellaris (n=45) AP: Anteroposterior; SD: standard deviation, Min: minimum, Max: maximum

	Mean±SD (mm)	Min-Max (mm)
Medial condyle AP length	59.573±3.88	50.69-66.39
Lateral condyle AP length	57.172±5.47	50.50-64.15
Sulcus height	51.186±3.81	42.28-57.58
Trochlear depth	7.436±1.19	4.60-9.51
Trochlear index	2.295±0.06	2.141-2.439
Facies patellaris length	29.893±4.41	21.27-40.38
Facies patellaris width	35.908±3.78	29.33-44.82
Facies patellaris medial articular surface	15.168±1.94	11.12-19.56
Facies patellaris lateral articular surface	20.733±2.50	15.64-25.26

The shape of the facies patellaris was classified as circular (37.8%), flat (17.8%), punctate (11.1%), triangular (31.1%), and triangular+punctate (2.2%), based on its border extending to the femoral corpus.

The groove called trochlea, situated on the facies patellaris, is not exactly in the middle, and the size of the articular surface extending along the medial and lateral aspects of the facies patellaris differs. Measurements revealed a smaller aspect ratio of the medial facet compared to the lateral facet in 44 dry bones. Only one dry bone had a larger medial articular surface (50.9%) compared to the lateral joint, which was different from the others. In 10 out of the 44 dry bones, the width of the medial articular surface was measured below 40% of the width of the facies patellaris (Table 3).

**Table 3:** Medial and lateral articular surface analysis of bones with a medial aspect ratio <40%

mm / %	1	2	3	4	5	6	7	8	9	10
Medial	11.12 (34.7%)	13.01 (39.5%)	13.67 (38.7%)	12.67 (39.8%)	14.40 (37.4%)	13.72 (37.1%)	11.98 (36.7%)	12.62 (39.3%)	14.27 (37.9%)	14.97 (39.3%)
Lateral	20.85 (65.3%)	19.90 (60.5%)	21.64 (61.3%)	19.10 (60.2%)	24.04 (62.6%)	23.70 (62.9%)	20.59 (63.3%)	19.47 (60.7%)	23.30 (62.1%)	23.06 (60.7%)

The measurements of the other 34 bones showed that the width of the medial articular surface was between 40% and 50% of the width of the facies patellaris, and the mean values of these bones are given in Table 4.

**Table 4:** Analysis of bones with a medial aspect ratio between 40% and 50%

	Medial articular surface	Lateral articular surface	Facies patellaris
Mean (mm)	15.69	20.65	36.34
Ratio (%)	43.1	56.8	100

A significant and strong positive correlation was found between trochlear depth and trochlear index values. The facies patellaris width was also found to be significantly positively correlated with both the trochlear depth (p=0.000) and facies patellaris length (p=0.002), indicating a strong and significant positive correlation with the aspect ratio of medial and lateral facets.

The facies patellaris length was found to be positively correlated with medial condyle AP length, sulcus height, facies patellaris width, and medial and lateral aspects of the joint. However, the correlation was statistically significant only for facies patellaris width (p=0.002), medial segment (p=0.025), and lateral segment of the joint (p=0.003).

An insignificant negative correlation was found between sulcus height and trochlear depth (p=0.536). However, the trochlear index was found to be negatively and significantly correlated with the sulcus height (p=0.000) and positively and significantly correlated with the trochlear depth (p=0.000). There was no statistically significant difference between the two researchers' measurements (p>0.05).

## Discussion

Understanding the morphology of the facies patellaris and other anatomical structures in the distal region of the femur is crucial before replacement surgery [1]. The significance of regional anatomy is also evident in the relationship between the patella and facies patellaris during knee joint motions and movements.

The facies patellaris features a groove called trochlea, and the patella plays a stability role in the anatomical position of the knee joint, closely interacting with the trochlea during flexion and extension. During knee joint movements, the patella glides on the facies patellaris, trochlea, and medial and lateral joint surfaces. Therefore, the anatomy of the region, including the trochlea, medial articular surface, and lateral articular surface, is crucial in the clinical evaluation of movements, especially in cases of trochlear dysplasia or patellar instability [10].

The mean medial condyle length in our study was 59.573±3.88 mm. Our data were comparable to those reported by Terzidis et al. (58.7±4.1mm) and Lakati et al. (58.01mm) [11,12]. It was somewhat lower than the 61±4.4 mm observed by Everhart et al. and the 63±5 mm recorded by Chandran et al. [13,14]. The mean lateral condyle length in our study was 57.172±5.47 mm, which is comparable to results reported by Nayak et al. (58.48±4.2 mm) and Terzidis et al. (58.5±4mm) [11,15]. Our data were higher than those reported by Khanal et al. (56±4.9mm) [16].

Various procedures have been described in the literature for trochleoplasty, with direct association with the anatomy of facies patellaris, trochlea, medial articular surface, and lateral articular surface [5,17]. Recognizing the anatomy of this region in different patient populations can guide clinicians in determining the indications and necessary interventions.

Furthermore, in countries like the Republic of Türkiye and other Asian cultures where people often keep their knees flexed and sit on the floor as part of their social and cultural lifestyle, the design of knee prostheses and their long-term compatibility should take these factors into consideration [7,18]. Therefore, understanding the differences in the relevant anatomical region, appropriate medical treatment, and selecting the most suitable implant can help prevent problems associated with the region.

Complications such as changes to the retropatellar region, abrasion, and degeneration have been described as potential outcomes of total knee arthroplasty [6]. It is important to ensure harmony between facies patellaris, trochlea, and patella to prevent an increase or decrease in pressure in the retropatellar space, which may occur after replacement surgery.

Limitations of the study

The study is limited to 45 dry bones (femurs) from the Faculty of Medicine, Department of Anatomy at Fırat University, with no regard for gender.

## Conclusions

The morphological data obtained from this study regarding the distal femur suggest that implant manufacturers should consider the anatomical characteristics of different ethnic groups and patient populations in their design. Based on these data, joint replacement manufacturers should address potential issues with implant compatibility, redesign implants as needed, and make suitable "anatomical" knee prostheses available. Determining anatomical differences in the patellar region, which is a crucial center of motion, is necessary not only to improve function but also to ensure long-term implant survival in a region with continued mobility.
